# α subunits in GABA_A_ receptors are dispensable for GABA and diazepam action

**DOI:** 10.1038/s41598-017-15628-7

**Published:** 2017-11-14

**Authors:** Nisa Wongsamitkul, Maria C. Maldifassi, Xenia Simeone, Roland Baur, Margot Ernst, Erwin Sigel

**Affiliations:** 1 Institute of Biochemistry and Molecular Medicine, University of Bern, Bern, Switzerland; 2 Department of Molecular Neurosciences, Center for Brain Research, Medical University of Vienna, Vienna, Austria; 3 Present Address: Centro Interdisciplinario de Neurociencia de Valparaíso. Facultad de Ciencias, Universidad de Valparaíso, Valparaíso, Chile

## Abstract

The major isoform of the GABA_A_ receptor is α_1_β_2_γ_2_. The binding sites for the agonist GABA are located at the β_2_+/α_1_− subunit interfaces and the modulatory site for benzodiazepines at α_1_+/γ_2_−. In the absence of α_1_ subunits, a receptor was formed that was gated by GABA and modulated by diazepam similarly. This indicates that alternative subunits can take over the role of the α_1_ subunits. Point mutations were introduced in β_2_ or γ_2_ subunits at positions homologous to α_1_− benzodiazepine binding and GABA binding positions, respectively. From this mutation work we conclude that the site for GABA is located at a β_2_+/β_2_− subunit interface and that the diazepam site is located at the β_2_+/γ_2_− subunit interface. Computational docking leads to a structural hypothesis attributing this non-canonical interaction to a binding mode nearly identical with the one at the α_1_+/γ_2_− interface. Thus, the β_2_ subunit can take over the role of the α_1_ subunit for the formation of both sites, its minus side for the GABA binding site and its plus side for the diazepam binding site.

## Introduction

γ-Aminobutyric acid type A (GABA_A_) receptors are the major inhibitory neurotransmitter receptors in the mammalian central nervous system. The GABA_A_ receptor is a pentameric protein complex, whose subunits are drawn from the following different isoforms: α(1–6), β(1–4), γ(1–3), δ, ε, θ, π and ρ(1–3). The five subunits form a chloride selective ion channel^1–3^. The most common isoform of this receptor consists of two α_1_, two β_2_ and one γ_2_ subunit(s)^4–6^ arranged α_1_γ_2_β_2_α_1_β_2_ counterclockwise when viewed from the extracellular space^7–9^. These receptors have two agonist GABA binding sites and one benzodiazepine binding site^10^. By using *in vitro* mutagenesis the binding sites for the agonist GABA were located to the β_2_+/α_1_− subunit interfaces^11,12^, and the modulatory site for benzodiazepines was at the α_1_+/γ_2_− subunit interface^13^. Thus, the α_1_ subunit is commonly accepted to contribute to the formation of both sites.

The GABA_A_ receptors can be activated by the agonist GABA and modulated by many drugs^14^. Among these drugs are the benzodiazepines, such as diazepam, that have sedative, anxiolytic, anticonvulsant, hypnotic, and muscle relaxant properties^15^. Coexpression of different combinations of recombinant subunits has generated GABA_A_ receptors with distinct pharmacological and electrophysiological properties.

As early as 1990, we observed that β_2_γ_2_ GABA_A_ receptors, lacking the α_1_ subunit, were activated by GABA and potentiated by diazepam^16^. Later this observation was confirmed by several groups for GABA and diazepam^17–19^ or other modulators^20^. Expression was also documented for β_1_γ_2_^21,22^ and β_3_γ_2_^22,23^ GABA_A_ receptors. In the present study, we tried to understand this apparent contradiction and decided to investigate whether alternative GABA and benzodiazepine-binding subunit interfaces exist. Site-directed mutagenesis was combined with two-electrode voltage clamp in *Xenopus* oocytes. Our findings suggest that the β_2_ subunit may replace the α_1_ subunit for the formation of either site. We have previously utilized experimentally guided computational docking that led to a diazepam bound structure model at the α_1_+/γ_2_− interface^24^. Computational docking at the β_2_+/γ_2_− interface yielded structural models which strongly suggest that diazepam can interact with this site in a binding mode nearly identical with the one observed at the canonical α_1_+/γ_2_− site, thus explaining the similar apparent potency.

## Results

### Functional expression of β_2_γ_2_ GABA_A_ receptors in *Xenopus* oocytes

We initially determined whether varying the subunit ratio led to a different extent of expression of β_2_γ_2_ GABA_A_ receptors. We injected β_2_ and γ_2_ cRNAs at the three ratios 1:1 (1 fMol each/oocyte), 2:1 (2 fMol and 1 fMol/oocyte) and 1:3 (1 fMol and 3 fMol/oocyte) into oocytes and measured the maximum current amplitudes elicited by 10 mM GABA. 5–7 days after microinjection of RNA, the β_2_γ_2_ GABA_A_ receptors formed by the 1:3 cRNA injection ratio gave the highest maximal current amplitude (131 ± 19 nA, n = 13). In contrast receptors formed from 1:1 and 2:1 cRNA ratios resulted in current amplitudes less than 100 nA. Thus, we used the 1:3 cRNA ratio coding for wild type or mutant β_2_ or γ_2_ subunits for all following experiments. Possibly the subunit arrangement is affected by the injection ratio as it has been documented in the case of β_3_γ_2_ GABA_A_ receptors^25^. Oocytes injected with 1 fMol coding for the β_2_ subunit only, or with 3 fMol coding for the γ_2_ subunit only, both did not result in current expression.

### β_2_γ_2_ receptors respond to GABA

**Figure 1:**
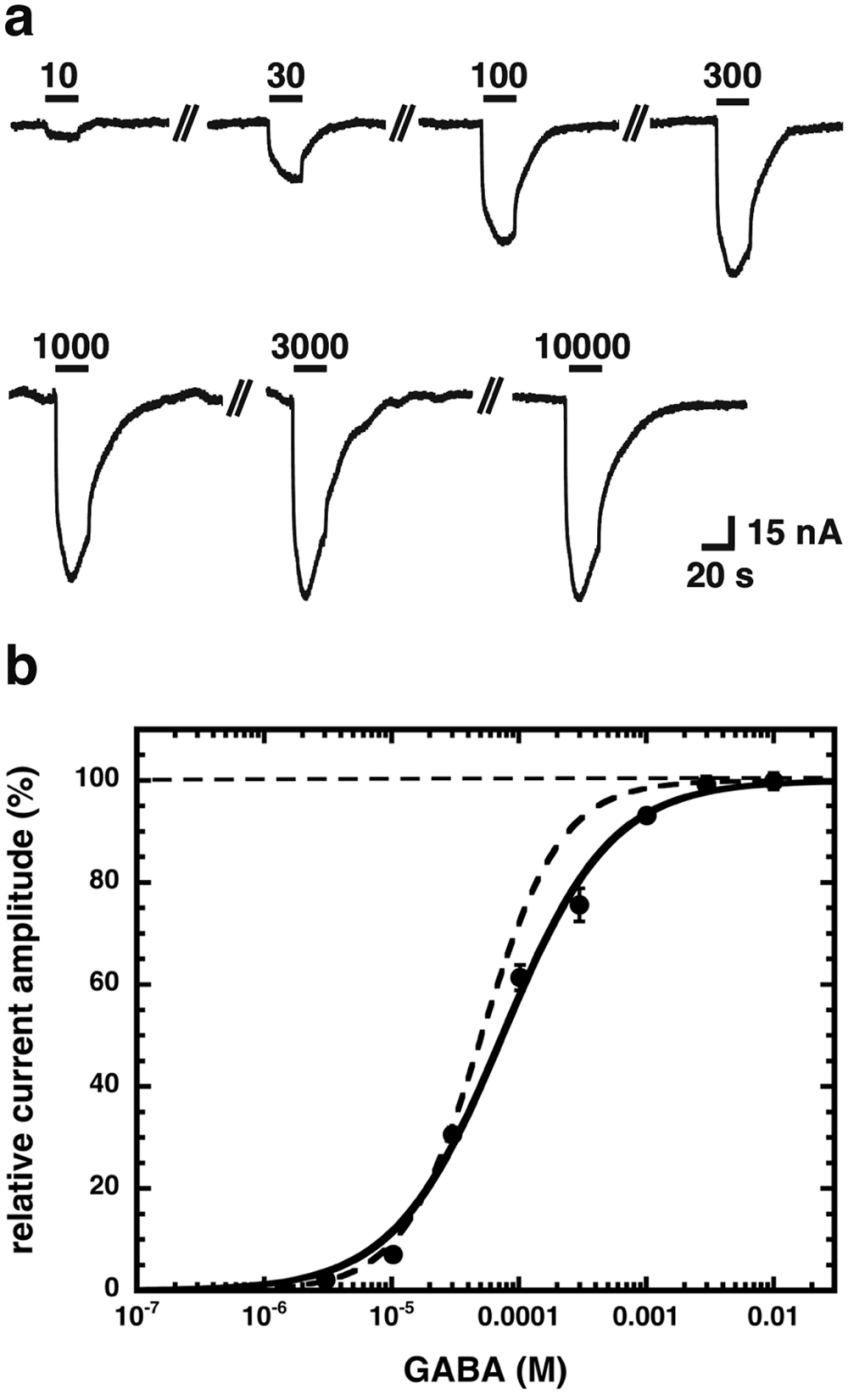
Concentration response curve for GABA at β_2_γ_2_ GABA_A_ receptors. Receptors were expressed in *Xenopus* oocytes and exposed to subsequently higher concentrations of GABA and the elicited current amplitude was determined. Individual curves were first normalized to the fitted maximal current amplitude and subsequently averaged. Data are expressed as mean ± S.E.M., n = 5 from two batches of oocytes. (**a**) Original current traces. GABA applications are indicated by a bar. The numbers indicate the concentration of GABA in μM. (**b**) Averaged concentration-response curve for β_2_γ_2_ GABA_A_ receptors. The dotted line shows for comparison corresponding data on α_1_β_2_γ_2_ GABA_A_ receptors.

5–7 days after injection, *Xenopus* oocytes expressing β_2_γ_2_ GABA_A_ receptors were investigated for the presence of currents elicited by 10 mM GABA. Figure 1a shows original current traces obtained from oocytes clamped at −80 mV. Figure 1b shows an averaged concentration-response curve for β_2_γ_2_ GABA_A_ receptors. The curve was characterized by an EC_50_ of 75 ± 5 µM and a Hill coefficient of 1.0 ± 0.1 (n = 5). This EC_50_ is similar to that reported for α_1_β_2_γ_2_, which amounts to 51 ± 15 µM^8^.

### β_2_γ_2_ receptors respond to diazepam

**Figure 2:**
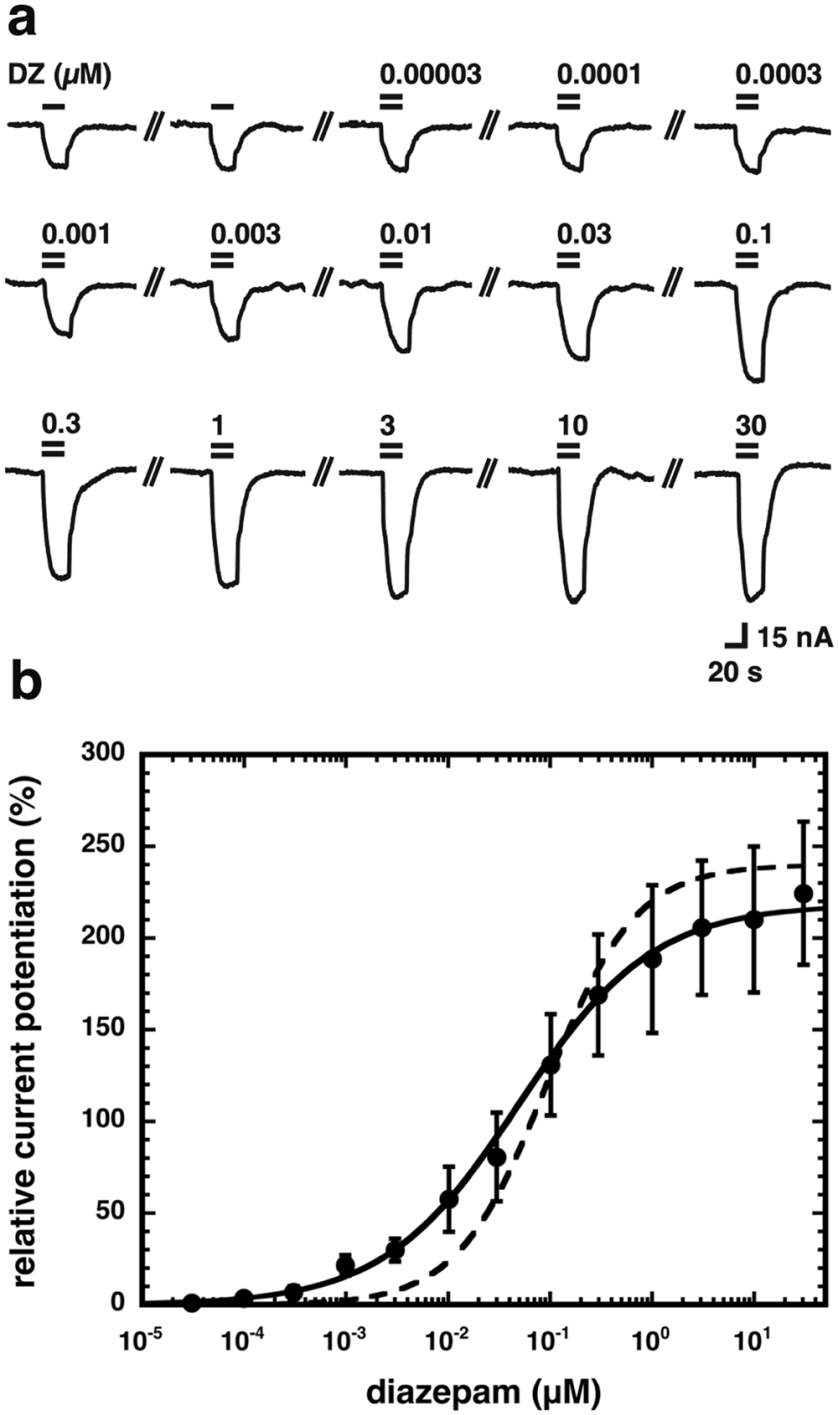
Concentration response curve for diazepam at β_2_γ_2_ GABA_A_ receptors. Receptors were expressed in *Xenopus* oocytes and exposed to either GABA alone or GABA in the presence of subsequently higher concentrations of diazepam and the elicited current amplitude was determined. At each concentration of diazepam current potentiation was calculated. Individual curves for potentation were first normalized to the fitted maximal current amplitude and subsequently averaged. Data are expressed as mean ± S.E.M., n = 3 from two batches of oocytes. (**a**) Original current traces. (**b**) Averaged concentration-response curve for β_2_γ_2_ GABA_A_ receptors. The dotted line shows for comparison corresponding data on α_1_β_2_γ_2_ GABA_A_ receptors.

Diazepam is a positive allosteric modulator of certain GABA_A_ receptors enhancing the GABA-induced chloride ion influx. We examined the current potentiation by 1 µM diazepam using a GABA concentration that elicited about 5% of the respective maximal current amplitude. Figure 2a shows original current traces from an experiment were oocytes were exposed to either GABA alone or in combination with increasing concentrations of diazepam. Figure 2b shows an averaged concentration-response curve. The curve was characterized by an EC_50_ of 69 ± 14 nM and a Hill coefficient of 0.6 ± 0.1 (n = 3). The EC_50_ is similar to that reported earlier for α_1_β_2_γ_2_ with 92 ± 6 nM^8^, while the Hill coefficient is, for reasons we do not understand, significantly lower than 1. No evidence for a possible receptor heterogeneity that could explain this finding was found (see below). Potentiation by 1 µM diazepam amounted to 216 ± 30% (n = 15).

### Selection of point mutations

**Figure 3:**
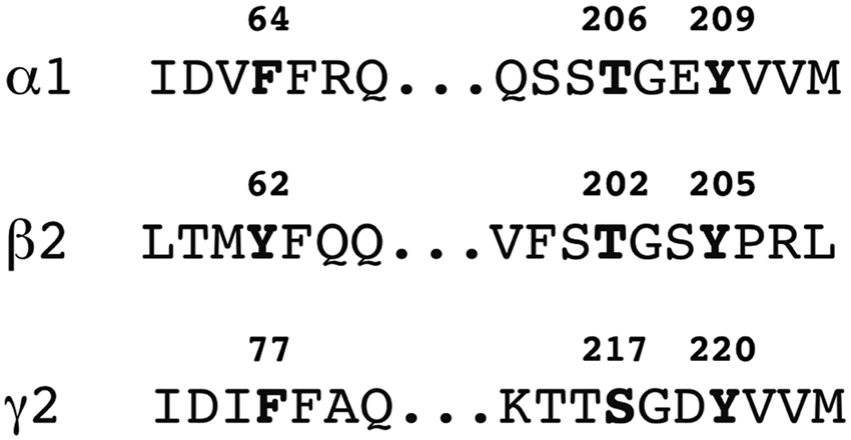
Sequence alignment of α_1_, β_2_ and γ_2_ subunits of the rat GABA_A_ receptor. Mutated residues of β_2_ and γ_2_ subunits are indicated with numbers. Homologous positions of the α_1_ subunit are also highlighted.

Obviously the α_1_ subunit is dispensable for the formation of a GABA_A_ receptor responsive to both channel agonist GABA and diazepam as shown above. We aimed to localize both binding sites in β_2_γ_2_ receptors. For this purpose we selected some point mutations that have been described to affect either the response to GABA or that to diazepam in α_1_β_2_γ_2_^11,12,26–29^ (Table 1). A total of nine different point mutations were introduced into either the β_2_ subunit or γ_2_ subunit. In the β_2_ subunit we generated one mutation at the minus side (β_2_Y62L) and four mutations at the plus side (β_2_T202A, β_2_T202S, β_2_Y205S, β_2_Y205Q) and in the γ_2_ subunit one mutation on the minus side (γ_2_F77Y) and three mutations on the plus side (γ_2_S217A, γ_2_Y220S and γ_2_Y220Q). A sequence alignment of the corresponding regions in α_1_, β_2_ and γ_2_ is shown in Fig. 3. Table 1 summarizes the consequences of the mutations in α_1_β_2_γ_2_ receptors. In addition the effect of homologous mutations in other subunits is listed.

**Table 1:** Point mutations in β_2_ and γ_2_ used in this study.

mutation	effect in α_1_β_2_γ_2_	homologous residues in α_1_ or β_2_	effect of homologous mutations in α_1_β_2_γ_2_	Lit.
β_2_Y62L	30-fold decrease in EC_50_ GABA	α_1_F64L	200-fold decrease in EC_50_ GABA	11
	no shift in antagonist apparent affinity		200-fold decrease in antagonist apparent affinity	
β_2_T202S	20-fold decrease in EC_50_ GABA	(α_1_T206C)	(5-fold increase in EC_50_ GABA)	12,27
			(no effect on DZ affinity)	
		(α_1_T206V)	(7-fold decrease in DZ affinity)	28
γ_2_F77Y	230-fold decrease in DZ affinity			29
	no effect on EC_50_ GABA			
γ_2_S217A	Not available	(β_2_T202A)	(drastic loss of EC_50_ GABA)	12
		(α_1_T206A)	(3-fold decrease in DZ affinity)	26
γ_2_Y220Q	Not available	α_1_Y209Q	see above	26

Point mutations were selected on the basis of previous findings in α_1_β_2_γ_2_ GABA_A_. The receptors β_2_T202Aγ_2_, β_2_Y205Sγ_2_, β_2_γ_2_Y220S and β_2_Y205Qγ_2_ were additionally studied, but were not activated by GABA or etomidate.

All mutated subunits were expressed in *Xenopus* oocytes in combination with wild-type subunits to result in β_2_γ_2_ GABA_A_ receptors. Functional properties were determined by using two-electrode voltage clamp.

Functional expression of receptors was verified using 50 µM etomidate as agonist since mutations affecting the response to GABA are not very likely to influence the response to this agent. Amino acid residues affecting the latter property have been described to be located within the membrane embedded part of the receptor^30^. The mutation β_2_Y62L interfered very strongly with channel activation by etomidate. As the mutation is located far away from the suspected etomidate site^31^, this indicates that this mutation also affects gating by etomidate (see below). Unfortunately, β_2_T202Aγ_2_, β_2_Y205Sγ_2_ and β_2_Y205Qγ_2_ all resulted in no or very little expression (Table 2), so that these receptors could not be further investigated.

**Table 2:** Functional properties of expressed wild type and mutated GAB_AA_ receptor subunits.

Subunit combination	Etomidate (nA; 50 µM)	GABA (nA; 10 mM)	EC_50_ (µM)	DZ potentiation (%)	Homology to mutated residues
β_2_	4 ± 1 (4)	−4 ± 1 (4)	—	—	—
γ_2_	0 ± 0 (5)	−4 ± 1 (5)	—	—	—
β_2_γ_2_	946 ± 8 (4)	131 ± 19 (13)	75 ± 5 (5)	216 ± 30 (15)	—
β_2_Y62Lγ_2_	13 ± 3 (11)	53 ± 8 (17)	228 ± 50 (7)	121 ± 12 (5)	α_1_F64, γ_2_F77
β_2_T202Aγ_2_	8 ± 2 (5)	5 ± 0 (5)	—	—	α_1_T206, γ_2_S217
β_2_T202Sγ_2_	380 ± 77 (5)	203 ± 18 (5)	3130 ± 260 (4)	100 ± 7 (4)	α_1_T206, γ_2_S217
β_2_Y205Sγ_2_	0 ± 0 (5)	0 ± 0 (16)	—	—	α_1_Y209, γ_2_Y220
β_2_Y205Qγ_2_	6 ± 2 (5)	1 ± 0 (4)	—	—	α_1_Y209, γ_2_Y220
γ_2_F77Yβ_2_	193 ± 25 (4)	148 ± 23 (11)	72 ± 15 (5)	5 ± 2 (5)	α_1_F64, β_2_Y62
γ_2_S217Aβ_2_	141 ± 31 (5)	100 ± 25 (13)	133 ± 20 (3)	185 ± 23 (4)	α_1_T206, β_2_T202
γ_2_Y220Sβ_2_	76 ± 17 (5)	10 ± 1 (12)	—	—	α_1_Y209, β_2_Y205
γ_2_Y220Qβ_2_	777 ± 113 (5)	127 ± 40 (6)	367 ± 139 (3)	127 ± 11 (6)	α_1_Y209, β_2_Y205

Individual subunits or subunit combinations were expressed in *Xenopus* oocytes. Assembly of receptors was verified by determining amplitudes of current responses to 50 µM etomidate. Responses to 10 mM GABA and potentiation by 1 µM diazepam were determined.

### Effect of a mutation at the plus side of β_2_

**Figure 4:**
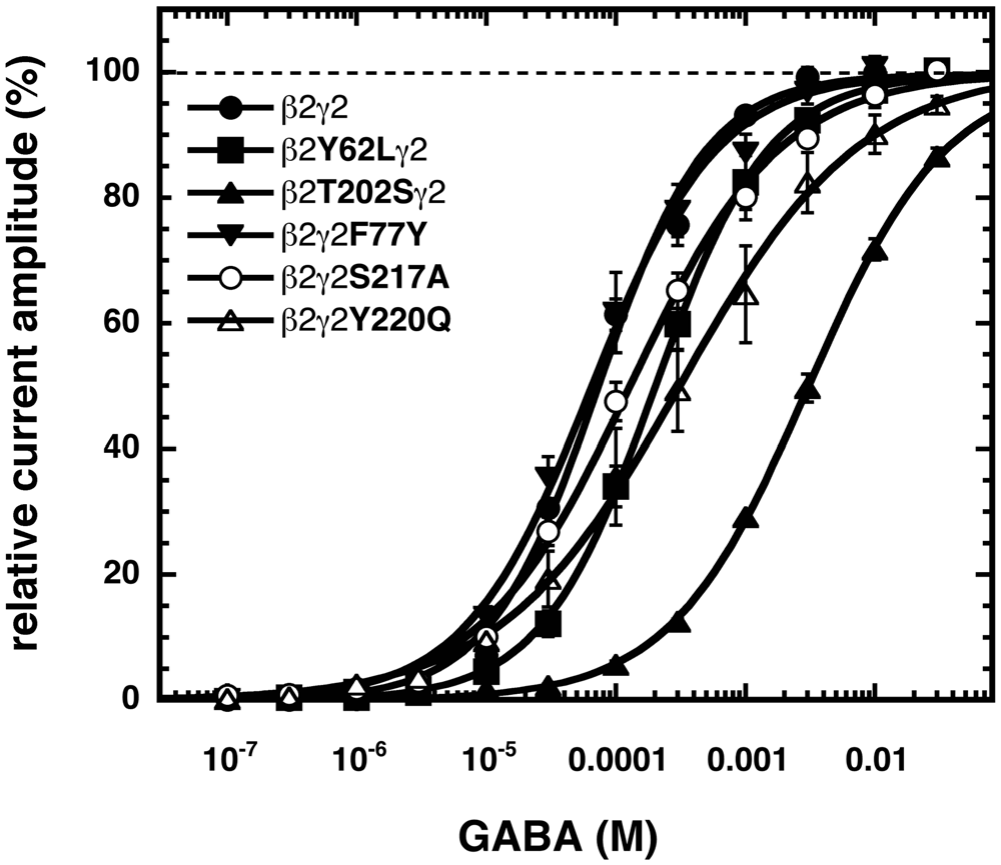
Influence of different point mutations on the concentration dependence of GABA. Wild type or mutant β_2_γ_2_ GABA_A_ receptors were expressed in *Xenopus* oocytes and exposed to subsequently higher concentrations of GABA and the elicited current amplitude was determined. Individual curves for each subunit combination were first normalized to the fitted maximal current amplitude and subsequently averaged. Averaged concentration-response curve are shown. Data are expressed as mean ± S.E.M., n = 3–7 from two batches of oocytes.

**Figure 5:**
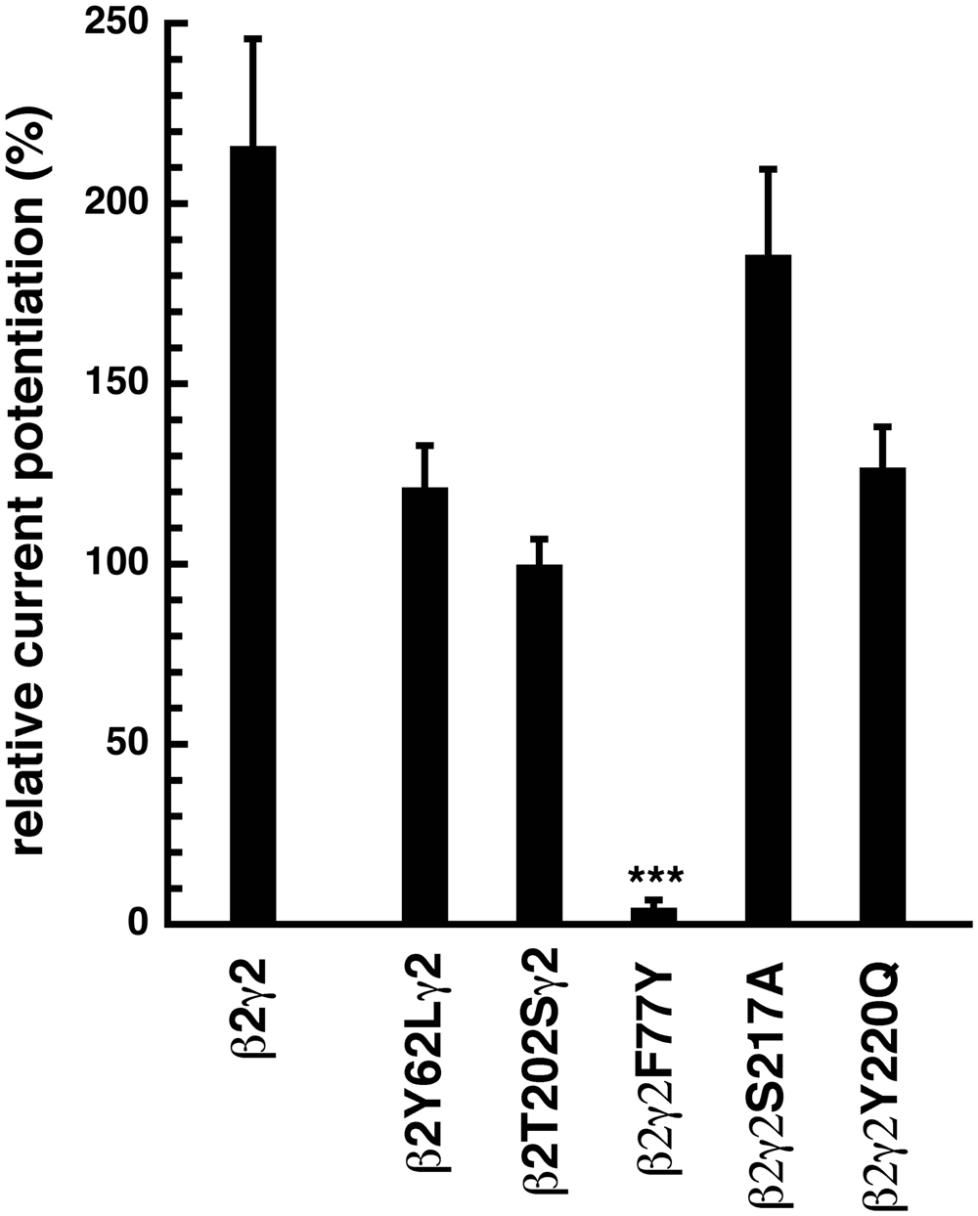
Influence of different point mutations on the potentiation by diazepam. Receptors were expressed in *Xenopus* oocytes and first exposed to 7 µM GABA alone or the same concentration of GABA in the presence of 1 µM diazepam and the elicited current amplitude was determined. Current potentiation by diazepam was calculated and averaged for each subunit combination. Data are expressed as mean ± S.E.M., n = 4–15 from two batches of oocytes.

β_2_T202S was the only investigated point mutation at the plus side of β_2_ that did not disrupt β_2_γ_2_ receptor assembly as evidenced by the large response to etomidate (Table 2). In α_1_β_2_γ_2_ receptors this mutation led to a 20-fold decrease in GABA sensitivity^12^. In β_2_γ_2_ receptors the same mutation led to a 42-fold decrease in GABA sensitivity (Fig. 4, Table 2). The EC_50_ for GABA dependent channel gating was 3130 ± 260 µM and a Hill coeffient of 0.8 ± 0.1 (n = 4). Potentiation by 1 µM diazepam was not significantly affected by the mutation (Fig. 5, Table 2).

### Effect of a mutation at the minus side of β_2_

In α_1_β_2_γ_2_ receptors the mutation β_2_Y62L leads to a 30-fold decrease in GABA sensitivity with no effect on the antagonist affinity as compared to wild-type α_1_β_2_γ_2_ GABA_A_ receptors^11^. The homologous mutation in the α_1_ subunit, α_1_F64L caused a 200-fold drop in both properties^11^. Here, we found that combination of β_2_Y62L with the γ_2_ subunit showed 2-fold smaller current amplitude (Table 2) as compared to wild-type β_2_γ_2_ receptors, indicating that this mutation might somehow disturb efficient assembly of functional channels and/or that gating is affected. In addition the mutation β_2_Y62L led to a 3-fold decrease in the sensitivity for GABA with an EC_50_ of 228 ± 50 µM and a Hill coefficient of 1.0 ± 0.1 (n = 7) (Fig. 4, Table 2). Again, potentiation by 1 µM diazepam was not significantly affected by the mutation (Fig. 5, Table 2).

The notion that gating is affected by the mutation is strongly supported by the fact that 50 µM etomidate elicits about 4-fold smaller currents than saturating concentrations of GABA (Table 2). In wild type β_2_γ_2_ receptors this current was about 7-fold larger than the one elicited by GABA. Similarly, in mutated α_1_β_2_Y62Lγ_2_ receptors this current was about 30-fold smaller than the one elicited by GABA. As the site for etomidate is located far away from the mutated residue, this is a strong indication that β_2_Y62 is not only involved in binding of GABA, but also in gating.

### Effect of mutations at the plus side of γ_2_

Among the investigated mutations, γ_2_S217A and γ_2_Y220Q led to sizeable expression in combination with β_2_ and were further studied. To our knowledge nothing is known about both mutations. The homologous mutation to γ_2_S217A in the β_2_ subunit, β_2_T202A, led to a drastic loss in GABA sensitivity^12^. The homologous mutation to γ_2_Y220Q in the α_1_ subunit, α_1_Y209Q, disrupted the site for diazepam, while leaving GABA sensitivity unaffected^26^. This residue was also identified with photoaffinity labeling by the benzodiazepine binding site ligand Ro15–4513^32^. In β_2_γ_2_S217A and β_2_γ_2_Y220Q the sensitivity to GABA was decreased 2-fold and 5-fold, respectively with EC_50s_ of 133 ± 20 µM (n = 3) and 367 ± 139 µM (n = 3), and Hill coefficients of 0.8 ± 0.1 and 0.7 ± 0.1, respectively (Fig. 4, Table 2). Potentiation by 1 µM diazepam was not significantly affected by both mutations (Fig. 5, Table 2).

### Effect of a mutation at the minus side of γ_2_

We studied the mutation γ_2_F77Y. In α_1_β_2_γ_2_ receptors this mutation abolishes the binding site for diazepam, while leaving GABA sensitivity unaffected^29^. Similar findings were made in β_2_γ_2_ receptors. The EC_50_ for GABA was 72 ± 15 µM (n = 5) and the Hill coefficient 0.9 ± 0.1 (Fig. 4, Table 2). Modulation by 1 µM diazepam was nearly lost (Fig. 5, Table 2).

### Summary of the findings

No appreciable currents could be elicited upon expression of β_2_ or γ_2_ subunits alone and the receptors β_2_T202Aγ_2_, β_2_Y205Sγ_2_, β_2_γ_2_Y220S and β_2_Y205Qγ_2_. Modulation by diazepam was nearly lost in β_2_γ_2_F77Y receptors, whereas the reponse to GABA remained unaffected. Activation by GABA was strongly affected in β_2_T202Sγ_2_ receptors and weakly affected in β_2_Y62Lγ_2_, β_2_γ_2_S217A and β_2_γ_2_Y220Q receptors.

### Computational Docking

We performed computational docking of diazepam utilizing a homology model of the β_2_+/γ_2_− interface based on the β_3_ crystal structure 4COF^33^ as specified in the Methods section. The overal sequence similarity between β_2_+ and α_1_+ is high, especially in loops B and C where several aromatic and polar amino acids are conserved. We have shown previously that loop C residues are engaged in key interactions with diazepam^24,26^. The docking as specified in the Methods section provided for sidechain flexibility (loops D, G, E, B and C,) as well as a limited degree of backbone flexibility in the loop C tip, very similar to the approach used in our previous docking studies at the canonical high affinity α_1_+/γ_2_− site^24^. Computational docking usually generates correct binding poses, but they are not always correctly ranked by the different scoring functions^34^. We therefore analyzed the top 100 poses of the docking run based on multiple different criteria: Only poses that display interactions with γ_2_F77 were considered, to limit poses to those that reflect experimental findings. Poses were then filtered by similarity to the binding mode in the high affinity α_1_+/γ_2_− site^24^, where similarity was judged on ligand binding mode and major interactions with the pocket. Lastly, two different scoring functions were employed to identify the best candidate poses based on consensus scoring. Overall, six poses were identified that show high similarity with the high affinity binding mode at α_1_+/γ_2_−. Among these, two were found in rank one and two positions in the ChemScore ranking, and five were among top 30 ChemScored. Similarly, one of the six candidates was found in the rank two position of the GoldScore Fitness ranking, and a total of three were among the GoldScore top 30. Thus, consensus scoring leads to a binding mode model that features a binding mode very similar to the one that is observed at the canonical high affinity site.

**Figure 6:**
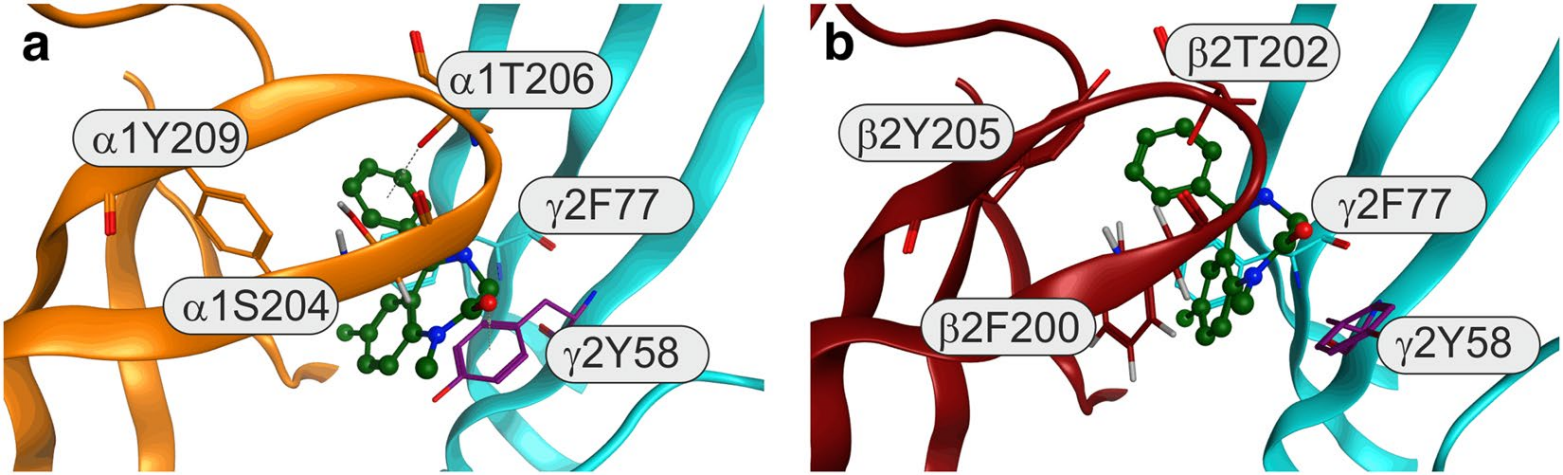
Structural hypothesis for diazepam binding at the extracellular β_2_+/γ_2_ interface. Panel (a) shows the reference binding pose from our previous studies^17^ at the α_1_+ (orange)/ γ_2_− (cyan) interface. Panel (b) shows the most closely corresponding binding pose from the computational docking at the β_2_+ (red)/γ_2_− (cyan) interface. The homologous key amino acids in the binding pockets, as well as diazepam, are rendered in stick representation. While sidechain rotamers show some differences, ligand position, binding mode and key interations are very similar.

Due to the sterically demanding sidechain β_2_F200, we find that sidechain rotamers adjust differently to ligand binding compared to the high affinity pocket, but gross binding mode and key interactions are highly similar (Fig. 6) where one of the representative poses is shown in comparison with the pose depicted in our previous study^24^.

In the proposed binding mode the pendant phenyl ring is in close contact to loop A, which bears in the diazepam sensitive α isoforms a histidine, and in α_4_ and α_6_ an arginine which is known to interfere with diazepam binding^35^. Several H101X mutations were investigated in the past, and it was demonstrated that F (Phe), Y (Tyr) or Q (Gln) have only a small impact on flunitrazepam modulation^36^. The homologous position in β_2_ is the hydrophobic Leu99, which is sterically similar to Gln, and as hydrophobic as Phe. Thus, the proposed binding mode is compatible with previous mutagenesis studies and with the present observations.

## Discussion

Several early studies reported responsiveness of β_2_γ_2_ GABA_A_ receptors expressed in heterologous systems to both GABA and diazepam^16–19^ or to GABA in β_x_γ_2_ GABA_A_ receptors^20–23^. Later, the two binding sites for GABA in α_1_β_2_γ_2_ GABA_A_ receptors were localized to the two β_2_+/α_1_− subunit interfaces^11,12^ and the diazepam binding site to α_1_+/γ_2_− subunit interface^13^. Thus, the α_1_ subunit seemed to be required for the formation of both sites. The major point of this study was to localize the subunit interfaces that harbor the alternative GABA and benzodiazepine binding sites in β_2_γ_2_ GABA_A_ receptors lacking the α_1_ subunit.

After having shown that β_2_γ_2_ GABA_A_ receptors responded similarly to GABA and diazepam as α_1_β_2_γ_2_ GABA_A_ receptors, we used point mutations abrogating one of the two in α_1_β_2_γ_2_. Unfortunately, some of the chosen mutations interfered with receptor expression or gating, presumably by negatively affecting assembly or by leading to mainly inactive channels.

### The γ_2_+/β_2_− subunit interface may be excluded for both sites

There are four possible subunit interfaces in β_2_γ_2_ GABA_A_ receptors: β_2_+/β_2_−, β_2_+/γ_2_−, γ_2_+/β_2_− and γ_2_+/γ_2_−. Of these, the γ_2_+/β_2_− subunit interface also occurs in the α_1_β_2_γ_2_ receptors. Mutation β_2_T202A dramatically impaired GABA activation in these receptors^12^ and mutation α_1_Y209Q led to loss of flumazenil sensitivity^26^. Obviously the γ_2_+/β_2_− subunit interface can not take over the formation of both sites. In addition three mutations located at this interface in β_2_γ_2_ receptors, β_2_Y62Lγ_2_, γ_2_S217Aβ_2_ and γ_2_Y220Qβ_2_, had no strong impact on the responses to GABA or diazepam (Table 2). Taken together, we can exclude that the γ_2_+/β_2_− subunit interface is the location of GABA and benzodiazepine binding sites.

### Localization of the diazepam binding subunit interface

Mutations β_2_T202S^12^ and γ_2_F77Y^29^ have been described to disrupt GABA and diazepam binding sites in α_1_β_2_γ_2_ receptors, respectively. Mutations β_2_T202S and γ_2_F77Y similarly strongly affect the response of β_2_γ_2_ receptors to GABA and diazepam. Mutations β_2_T202S and γ_2_F77Y had little impact on diazepam and GABA sites, respectively. This implies a role of β_2_+ and γ_2_− in the formation of GABA site and diazepam site, respectively. Thus, the GABA binding site must be located at β_2_+/β_2_− or β_2_+/γ_2_− subunit interfaces and that for diazepam at β_2_+/γ_2_− or γ_2_+/γ_2_−.

**Figure 7:**
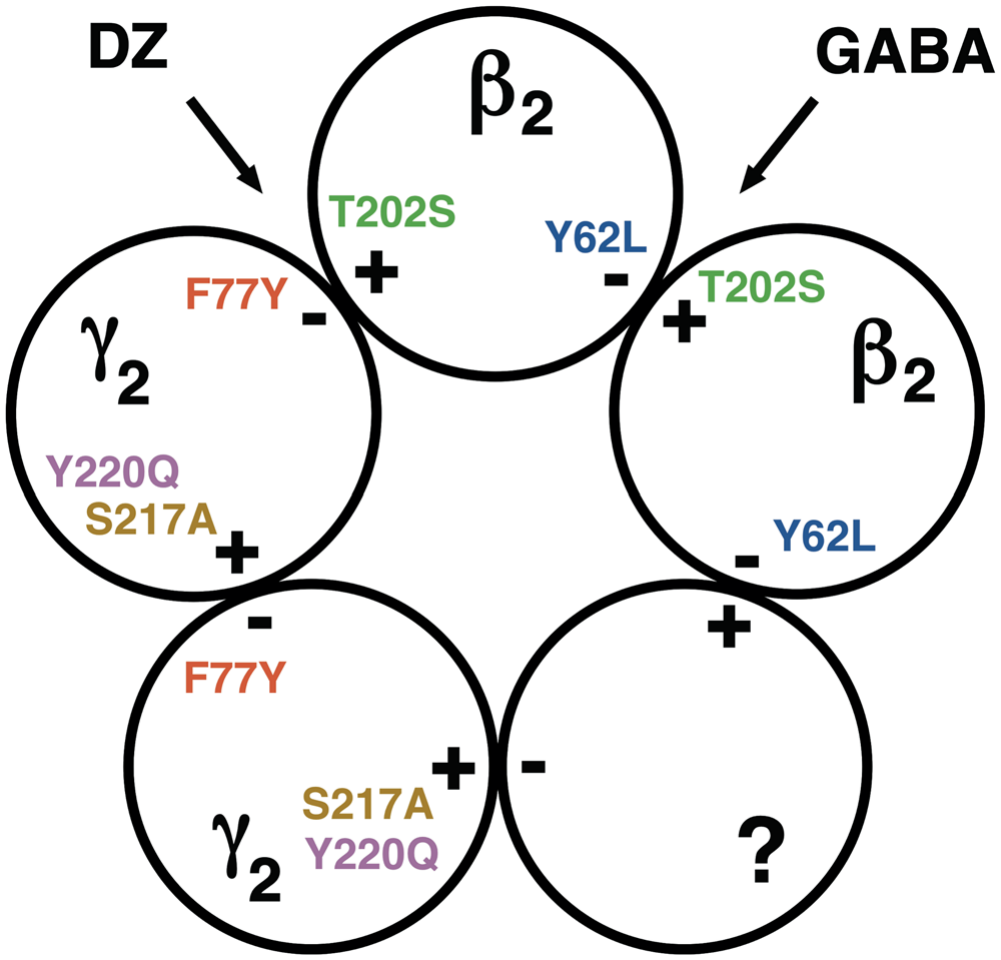
Schematic representation of β_2_γ_2_ GABA_A_ receptors. The location of amino acid residues of interest is indicated. Point mutations resulting in disrupting assembly are not shown. The binding site for GABA is concluded to locate at the β_2_+/ β_2_− interface, that for diazepam at the β_2_+/γ_2_− interface. Please note that the subunit arrangement was not addressed in this study.

The mutation α_1_Y209Q abrogates the diazepam site in α_1_β_2_γ_2_ receptors^26^. Therefore, it may be expected that the homologous mutation in the γ_2_ subunit, γ_2_Y220Q, affects the apparent affinity for diazepam. Similarly, the mutation α_1_T206A decreases the affinity for diazepam in α_1_β_2_γ_2_ receptors^26^. Thus, it may be expected that the homologous mutation in the γ_2_ subunit, γ_2_S217A, affects the apparent affinity for diazepam. Both mutations failed to affect the response to diazepam, arguing strongly against involvement of the γ_2_+ subunit interface in the diazepam site. For these reasons, we locate this site to the β_2_+/γ_2_− subunit interface (Fig. 7). The failure of the mutation β_2_T202S to affect the response to diazepam may be explained by the fact that a homologous, similar mutation α_1_T206C did not affect the response to diazepam in α_1_β_2_γ_2_ receptors^27^. The high affinity interaction of diazepam with the β_2_+/γ_2_− subunit interface is also supported by the docking experiments (Fig. 6), which strongly suggest that diazepam binds in this non-canonical site in a fashion very similar to the one that is observed in the high affinity site.

### Putative localization of the GABA binding subunit interface

Thus, we are left with the β_2_+/β_2_− and the γ_2_+/γ_2_− subunit interfaces. The mutation β_2_T202S at the β_2_+ has very strong effect on the EC_50_ for GABA shifting the concentration response curve 42-fold, while effects of mutations at the γ_2_+ side are much smaller. Therefore we localize the GABA binding site at the β_2_+/β_2_− subunit interface (Fig. 7). However, we can not fully exclude an additional site at a γ_2_+ side. As β_2_+/γ_2_− has been excluded, this additional GABA site would have to be at the γ_2_+/γ_2_− interface. It should be noted that a binding site for GABA has also been described at the β_3_+/δ− subunit interface^37^.

### Functional expression

We observed that individual β_2_ or γ_2_ subunits did not form functional receptors on the surface of oocytes. Within 1 day after injection, the α_1_β_2_γ_2_ receptors expressed currents in µA range^38^. In contrast, we observed in this work that the β_2_γ_2_ receptors need longer period for channel expession (5–7 days) and the maximal current amplitudes elicited by 10 mM GABA amount to only 100–200 nA. If silent receptors and different single channel open frequency between receptors are ignored, β_2_γ_2_ receptors form less efficiently than α_1_β_2_γ_2_ receptors. A previous study described a important role of α_1_ subunits for receptor trafficking and assembly in α_1_β_2_γ_2_ receptors^39^. Thus, in the presence of large amounts of α_1_, β_2_γ_2_ may not be formed. Under special circumstances where α subunit expression is low, the formation of a limited amount of β_2_γ_2_ receptors may occur. Recent single cell RT-PCR data indicate that cells devoid of mRNA coding for α subunits are not present in the hypothalamus, where diversity of neurons is huge. However, the endocrine system may have receptors without α subunits. Chromaffine cells (at least in certain developmental stages) have only mRNA coding for β_3_ and ε subunits (personal communication, I. Adameyko).

While so far it is not considered a candidate receptor to exist in the adult mammalian nervous system, the possible existence of such receptors has also not been specifically excluded. Given that fact that in the developing mammalian brain expression of all three γ isoforms is higher than in the postnatal brain, non-canonical receptor arrangements should be considered. In this vein, it is very important to realize that a high affinity benzodiazepine binding site at the β_2_+/γ_2_− interface implies that such receptors cannot be distinguished from α+/γ_2_− “canonical” receptors in radioligand and PET studies where benzodiazepine ligands are used as presumably selective probes for α+/γ_2_− canonical benzodiazepine-sites.

What may be the biological relevance of our observations? Ralvenius *et al*.^40^ studied mice carrying a point mutation in all those four alpha subunits that can form diazepam sensitive GABA_A_ receptors. At 10 mg/kg diazepam these mice were completely protected from diazepam-induced muscle relaxation and motor impairment. However, they showed a trend towards reduced locomotor activity that was quite prominent at higher doses. At least part of this response could be due to β_2_γ_2_ GABA_A_ receptors. We have not tested whether β_1_ or β_3_ (that may form β_1_γ_2_ and β_3_γ_2_) behave as β_2_. As their loop C differs (see Supplementary Fig. S1), it is conceivable that the diazepam site described here does not exist or has different properties in these receptors.

### Summary

While in α_1_β_2_γ_2_ receptors diazepam binds to the α_1_+/γ_2_− subunit interface and GABA to β_2_+/α_1_−, in β_2_γ_2_ receptors diazepam binds to the β_2_+/γ_2_− subunit interface and GABA to β_2_+/β_2_− (Fig. 7). Thus, the β_2_ subunit can take over the role of the α_1_ subunit for the formation of both sites, its minus side for the GABA binding site and its plus side for the diazepam binding site.

## Methods

### Construction of mutated receptor subunits

The point mutations β_2_Y62Lγ_2_, β_2_T202Aγ_2_, β_2_T202Sγ_2_, β_2_Y205Sγ_2_, β_2_Y205Qγ_2_, β_2_γ_2_F77Y, β_2_γ_2_S217A, β_2_γ_2_Y220S and β_2_γ_2_Y220Q were prepared using the QuickChange^TM^ mutagenesis kit (Stratagene, Agilent Technologies, Basel, Switzerland).

### Expression in *Xenopus* oocytes

Animal experiments were carried out in strict accordance to the Swiss ethical guidelines, and have been approved by the local committee of the Canton Bern Kantonstierarzt, Kantonaler Veterinärdienst Bern (BE85/15). Surgery of *Xenopus laevis* to obtain the oocytes was done under anesthesia, and all efforts were made to diminish animal suffering. Oocytes were prepared, injected and defolliculated as described previously^41,42^. Polyadenylated cRNA coding for the subunits of GABA_A_ receptors were prepared *in vitro* with the mMESSAGE mMACHINE kit (Ambion, Austin, TX, USA). Oocytes were injected with 50 nl of solution containing cRNA coding for wild type or mutants β_2_ (1 fMol) or γ_2_ (3 fMol) subunits and then incubated in modified Barth’s solution (10 mM HEPES, pH 7.5, 88 mM NaCl, 1 mM KCl, 2.4 mM NaHCO_3_, 0.82 mM MgSO_4_, 0.34 mM Ca(NO_3_)_2_, 0.41 mM CaCl_2_, 100 units/ml penicillin, 100 µg/ml streptomycin) at 18 °C for 5–7 days before measurements.

### Functional characterization in *Xenopus* oocytes

Electrophysiological experiments were performed using an Oocyte Clamp OC-725 (Warner Instrument Corp., Hamden, USA) two-electrode voltage clamp amplifier. Currents were digitized at 5 kHz with MacLab/200 (AD Instruments, Spechbach, Germany).

The holding potential was −80 mV. The perfusion medium contained 90 mM NaCl, 1 mM KCl, 1 mM MgCl_2_, 1 mM CaCl_2_ and 5 mM Na-HEPES (pH 7.4). The perfusion solution (6 ml/min) was applied through a glass capillary with an inner diameter of 1.35 mm, the mouth of which was placed about 0.5 mm from the surface of the oocyte. Individual concentration response curves for GABA were fitted with the equation I(c) = I_max_/[1 + (EC_50_/c)^n^], where c is the concentration of GABA, EC_50_ the concentration of GABA eliciting half-maximal current amplitude, I_max_ is the maximal current amplitude, I is the current amplitude, and n is the Hill coefficient. Maximal current amplitudes (I_max_) were obtained from the fits of the concentration-response curves. The individual curves were fitted and standardized to I_max_ and subsequently averaged. For all receptors studied, potentiation was measured at a GABA concentration eliciting 1–5% of the maximal GABA current amplitude. GABA was applied twice alone for 20 s, and then in combination with diazepam for 20 s. The duration of washout periods was 4 min in between agonist or agonist/drug applications to prevent receptor desensitization. At the beginning of the experiments, GABA applications were repeated when the elicited current amplitude altered by >5%. Potentiation was calculated by the following equation: (I_Modulator + GABA_/I_GABA_−1) * 100%. Concentration dependent potentiation was fitted with the equation I(c) = I_max_/[1 + (EC_50_/c)^n^], where c is the concentration of diazepam, EC_50_ the concentration of diazepam eliciting half-maximal current amplitude, I_max_ is the maximal current amplitude, I is the current amplitude, and n is the Hill coefficient. Maximal current amplitudes (I_max_) were obtained from the fits of the concentration-response curves. The individual curves were fitted and standardized to I_max_ and subsequently averaged.

All data are from at least two different batches of oocytes. Data represent mean ± S.E.M as indicated in each case. An unpaired *t-test* was used to compare two means. ***p < 0.001.

### Computational Modelling and Docking

Homology models of the β_2_+/γ_2_− interface were generated based on the 4COF GABA_A_ receptors human β_3_ homopentamer structure^24^. Due to the high homology of the β_2_ and β_3_ subunits, no insertions or deletions requiring gaps occur in the extracellular domain, while an alignment of the γ_2_ subunit as described previously^43^ was used to account for the lower homology in the loop F region. Computational docking was subsequently performed using the GOLD software v1.6.2^44^, where the binding site was defined to be at the interface between loops A – G of the subunits. Sidechains β_2_Y157, β_2_T160, β_2_Y159, β_2_T161, β_2_F200, β_2_T202, and β_2_Y205 as well as γ_2_Y58, γ_2_F77 and γ_2_T142 were kept flexible, soft potential were applied to the tip of loop C (β_2_V198 - β_2_G203) to allow some degree of backbone flexibility, default settings for the docking run were used and the top 100 ranked poses were retained for subsequent analysis. Ligand interactions of poses were computed using the MOE program (MOE (The Molecular Operating Environment), Version 2011.10, Chemical Computing Group Inc., Montreal), and poses featuring interactions with γ_2_F77 were inspected using two scoring functions (ChemScore Fitness as implemented in GOLD, GoldScore Fitness). “Hits” were defined as poses among top 30 ranked in both scoring functions (consensus score hits) and featuring interactions with γ_2_F77 and the resulting hit poses were subsequently compared to our poses from previous work at the canonical binding site^24^.

### Data availability

The datasets generated during and/or analysed during the current study are available from the corresponding author on reasonable request.

## Electronic supplementary material

Supplementary Information
