# Supplementary material for: α subunits in GABAA receptors are dispensable for GABA and diazepam action
Source: Sci Rep. 2017 Nov 14;7:15498. doi: 10.1038/s41598-017-15628-7 (PMC5686171; doi:10.1038/s41598-017-15628-7)
Supplement: Supplementary file 1 — Supplementary Information [file 41598_2017_15628_MOESM1_ESM.pdf]

Supplementary information

α subunits in $GABA_A$ receptors are dispensable for GABA and diazepam action

Nisa Wongsamitkul[1], Maria C. Maldifassi[1,2], Xenia Simeone[3], Roland Baur[1], Margot Ernst[3], Erwin Sigel[1,*]

[1]Institute of Biochemistry and Molecular Medicine, University of Bern, Bern, Switzerland

[2]present address: Centro Interdisciplinario de Neurociencia de Valparaíso. Facultad de Ciencias, Universidad de Valparaíso, Valparaíso, Chile.

[3]Department of Molecular Neurosciences, Center for Brain Research, Medical University of Vienna, Vienna, Austria

Supplementary Figure S1

Comparison of loop C in α_1, β_1, β_2 and β_3:

α_1	GIVQSSTGEY
β_2	KKVVFSTGSY
β_1	KKVEFTTGAY
β_3	RNVVFATGAY
